# Supratentorial Pediatric Midline Tumors and Tumor-like Lesions: Clinical Spectrum, Natural History and Treatment Options

**DOI:** 10.3390/children9040534

**Published:** 2022-04-09

**Authors:** Luca Paun, Alexandre Lavé, Gildas Patet, Andrea Bartoli

**Affiliations:** Division of Neurosurgery, Department of Clinical Neurosciences, Geneva University Hospitals, 1205 Geneva, Switzerland; alexandre.lave@hcuge.ch (A.L.); gildas.patet@hcuge.ch (G.P.); andrea.bartoli@hcuge.ch (A.B.)

**Keywords:** Sellar tumor, colloid cyst, pineal gland region, third ventricle, incidentaloma, hydrocephalus

## Abstract

Childhood Central Nervous System tumors account for 25% of all pediatric tumors. Large availability and broadening of indications to imaging has made incidental findings more common. Among these, midline lesions have different clinical relevance depending on their intrinsic pattern of behaviour and on their specific location. In this narrative review we describe the natural history and treatment options of midline lesions in children.

## 1. Introduction

Childhood Central Nervous System Tumors (CCNST) account for 25% of all childhood tumors and they represent the most common type of cancer after blood cancer. Although surgery, chemotherapy and radiation treatment improved overall survival (OS), CCNST are still the leading cause of cancer mortality in this population [1,2]. Among those tumors, midline lesions are heterogeneous and include pineal and third-ventricle associated tumors (3–11% of all CCNST) [3,4], optic gliomas (3–7%) [5,6] and sellar lesion (0.5–6%) [7]. Even if rare, midline lesions prevalence is growing thanks to imaging availability and novel diagnostic techniques, augmenting incidentalomas prevalence in the pediatric population [8,9,10,11,12]. On a large cohort of 3966 Magnetic Resonance Imaging (MRI) at least one incidental finding was present in 25.6% of the pediatric population [13]. The most common findings were pineal cysts (PC) (16.8%), arachnoid cysts (2.17%) and developmental venous abnormalities (1.59%) [13]. While some can show benign clinical behaviour being essentially asymptomatic over a life span, others can become symptomatic. Their midline location accounts for specific clinical patterns of symptoms, typically in four different ways: elevated intracranial pressure from obstructive hydrocephalus (HCP), neuroendocrine dysfunction, visual deficit and neuropsychological-behavioural changes. Clinical relevance and subsequent management options of pediatric midline lesions are based on this set of symptoms, imaging characteristics, location and dimensional relationship to adjacent structures [14]. In this review we summarize and define the most common midline lesions in the pediatric population, their clinical relevance and management options.

## 2. Clinically Benign Lesions

### 2.1. Rathke Cleft Cyst

Rathke cleft cysts are rare, mostly discovered as an incidental finding and they are more common in females (79%) [15,16]. They share a common embryonic origin with craniopharyngiomas even though their clinical behaviour is usually asymptomatic [17]. They typically lack of calcifications on Computed Tomography (CT) scan and they show a cystic component of various intensities on MRI sequences (depending on the mucoid material of its content), without solid enhancement. When the cystic component within the sellar region shows the same signal than Cerebrospinal Fluid (CSF), alternative differential diagnosis includes arachnoid cysts. The majority of cases are asymptomatic (66–80%) and clinical and radiological follow up can be proposed, especially when discovered incidentally [16]. Symptomatic cases are rare and they manifest with visual deficits, headaches and pituitary dysfunction. Post-operative endocrinopathies are common, especially diabetes insipidus (19–22%). Recurrence incidence is 11–14%, but further studies should be undertaken to define it on long term [16]. Metaplasia on the cyst wall, size and incomplete resection increase the risk of recurrency [18].

### 2.2. Arachnoid Cyst

Arachnoid cysts seem to arise from developmental abnormality, such as incompetence of the sellar diaphragm, splitting or duplication of arachnoid membrane, but they can also arise de novo after head traumas or intraventricular hemorrhage and are usually asymptomatic [19]. Their natural history is still not well understood, but it is thought that osmotic gradient, slit valve mechanism or CSF production by cyst wall can participate in enlargement process [19]. They are more common in male with an incidence peak before 2 years old and with a 2.6% prevalence [20]. 6% can rupture or develop a hemorrhage: this risk is augmented by larger cyst size and recent head trauma [21]. These tumor-like lesions can be followed on imaging and endocrine workup unless they become symptomatic on visual function, where surgery becomes the first line treatment [22]. If symptomatic, cyst fenestration can be proposed. Risk factors for developing symptoms are lower age, male sex and multiple cysts. In different series, mean overall treatment success rate is 3.6–15.6%, with long term post-operative neurocognitive improvement [19]. In asymptomatic cases, initial follow up is suggested for at least 3 years: if size stays stable there is a high rate of unchanging dimensions. Older age at the presentation is associated to radioclinical stability in time [19,20].

### 2.3. Colloid Cyst

Colloid cysts represent 0.5–1% of intracranial lesions and 55% of third ventricle lesions [23,24,25]. Male patients are more affected and colloid cysts tend to be located in the anterosuperior part of the third ventricle, probably originating from the diencephalic vesicle or from the persistence of the embryonic paraphysis. Their growth is extremely slow and they are found either as incidental finding or when they cause obstruction of the foramina of Monro and subsequent biventricular HCP. On imaging they are hyperintense on T1 and hypointense on T2 depending on their proteinaceous content. Incidence of symptomatic progression is between 0–11.2% at 10 years while 6% show radiological progression without symptoms, allowing for a regular follow up until the risk of HCP is deemed important [26,27,28]. Radiological follow-up is essential to monitor size changing and potential new symptoms [29]. When symptomatic, resective surgery should be offered, considering a 34% risk of acute deterioration without treatment [25]. 

On the other hand, even if asymptomatic, colloid cysts with irregular shape should be seen by a specialist [25]. If surgical intervention is considered and done, HCP symptoms usually resolve [29]. Exception is done for cases associated with aqueductal stenosis, where additional CSF diversion should be performed.

### 2.4. Pineal Cyst

PC are benign lesions, they can be considered an anatomical variation and they are more common in young females with a prevalence of 2% [10]. Considering the widespread use of neuroimaging the incidence of PC seems higher when compared to the past, leading patients and family to psychological and physical stress [8,30]. On a large cohort of incidental findings on brain imaging in general pediatric population, the most common findings were PC (16.8%) [31]. Different theories exist about PC formation: some argue that they are the result of a focal degeneration of pineal gland while others suggest that they originate from the proliferation of the wall of third ventricle into the diencefalic roof [32,33]. At MRI they have a spherical-oval shape and they can be found within other types of lesions mentioned above. MRI is the gold standard examination for this lesion where septation within the cystic cavity can be demonstrated [34]. A large majority is diagnosed as incidentalomas and normally PC remain asymptomatic without clinical consequences [35,36]. Natural history of these lesions is still debated, but it was shown that female patients older than 10 with higher active estrogen levels are at higher risk for PC progression [9,29]. The most common clinical presentation is chronic headache with subtle onset. Other symptoms are nausea, vomiting, gaze and gait problems and Parinaud’s syndrome considering their anatomical proximity to the tectal plate. HCP is the most dangerous complication together with cyst apoplexy (rare but potentially life-threatening) [37]. There seems to be no direct correlation between size and symptoms, even though the majority of PC become symptomatic when larger than 20 mm [33]. 

Their clinical management is tailored and is based on PC dimension, symptomatology and psychophysical stress. Considering that most of the PC are incidental findings and require no treatment, patient’s family should see a specialist to be reassured about the natural history of those lesions. Al Houlou et al. proposed a strict clinic-radiological follow-up until the adult age, when it can be widened and eventually stopped [38]. The same approach was proposed by Arkar et al., adding a special follow-up if the diameter is larger than 20 mm or if signs of cranial hypertension are seen at MRI [11]. Surgery is considered for obvious size progression or if new symptoms appear and are clearly related to the progression (paroxysmal headaches and gaze palsy; chronic headaches, papilledema and HCP; pineal apoplexy with acute HCP) [3].

## 3. Not Clinically Benign Lesions

### 3.1. Sellar Tumors

Sellar tumors include mainly craniopharyngiomas and pituitary adenomas. When these findings are unrelated to HCP, visual loss or endocrine/neuropsychological deficits, they can be considered incidental findings. 

#### 3.1.1. Craniopharyngioma

They are the most common tumor in the pituitary fossa in children (80–90% of all pituitary tumors) [39] and they typically present in childhood or during the fifth decade [40], rarely being an incidental finding. Following 2021 World Health Organization (WHO) Central Nervous System (CNS) classification they can be distinguished between the adamantinomatous type (presenting a CTNNB1 gene alteration) and the papillary type (BRAF-altered) [41]. 

While its biological and histological characteristics can be considered benign, its clinical behaviour can be devastating, notably where there is hypothalamic invasion and visual loss. Most frequent symptoms are headaches, visual deficits, endocrine deficits (growth impairment, weight gain, polydipsia-polyuria) or raised intracranial pressure from HCP [42]. Typical radiological findings on MRI or CT scans include enhancement, cystic component and calcifications [43]. Clinical workup must include assessments of hypothalamic-pituitary function and visual function. Current general approach showing good long-term disease control and survival rate include surgical resection sparing neurovascular and hypothalamic structures followed by radiotherapy [44,45]. Disease control can widely range between 44 and 93%, with an OS at 5-to-10 years ranging from 85 to 92%: As shown by different studies the aim of the treatment should focus on quality of life rather than on gross tumor resection (GTR) [46,47]. Postoperative endocrine function is still poor with a high rate of permanent hypopituitarism, obesity and diabetes insipidus. Further studies on long-term endocrine function management should be undertaken [47]. 

#### 3.1.2. Pituitary Adenoma

Very rare in children, pituitary adenomas account for less than 3% of all intracranial tumors under 20 years of age. They are diagnosed as incidentalomas with an incidence rate of 257/100.000 patients/year [48,49,50]. 51.2% are identified on scans for non-specific headaches [50]. When compared to adults, pediatric pituitary adenomas are typically functioning (80–97%) (Adrenocorticotropic Hormone (ACTH)-secreting, Prolactine (PRL)-secreting and Growth Hormone (GH)-secreting) and less frequently non-functioning [51,52]. They can be sporadic or less frequently part of a genetic syndrome (i.e., Multiple Endocrine Neoplasia (MEN)-1, McCune-Albright, Familial Isolated Pituitary Adenomas) [53].

When present, clinical findings and endocrine dysfunction will depend on the secreting hormone. On contrast T1 MRI of the pituitary gland, adenomas enhances less and in a delayed fashion than normal pituitary parenchyma [53]. Management of pituitary adenomas varies, depending on the clinical history (incidental vs. symptomatic), endocrine abnormalities when present, and mass effect on neurovascular structures.

ACTH secreting adenomas are the most common pituitary adenomas in children [54] and they are typically smaller (<3 mm) than other adenomas. They account for 98% of pediatric cases of Cushing’s disease and they are more common in males then females [55]. Differential diagnosis must include primary adrenal tumors, ectopic ACTH or Corticotropin-releasing Hormone (CRH) production and it is based on specific pediatric endocrine work-up that includes cortisol-suppression and ACTH and cortisol stimulation tests [56]. First treatment option is ideally surgery, with acceptable curing rate [57,58]. When not feasible or when recurrent, radiation therapy can be offered with good normalization of cortisol levels [59] but higher risk for long life hormonal replacement therapy [56].

Prolactinomas are the second most common pituitary adenomas with symptoms onset in late childhood [55]. Compared to ACTH secreting adenomas, prolactinomas are more commonly occurring in MEN-1 and AIP-mutated syndromes. In the diagnostic process, all other causes of hyperprolactinaemia should be ruled out and ultimate laboratory diagnosis is made considering persistent hyperprolactinemia compared to mean age and sex. Blood basal PRL correlates with the size of the lesion and it has high diagnostic value [55,60]. First treatment option for prolactinomas is usually based on dopamine-agonists, unless there is clear acute visual impairment or HCP [55]. Surgery and/or radiotherapy are considered as well for prolactinomas refractory to medical treatment [61].

GH secreting adenomas account for 5–15% of all adenomas, with a higher incidence in males and a median symptoms onset at 9 years old [55]. GH secreting adenomas may present in 36% of cases with typical gigantism, an acceleration of growth velocity (when the hypersecretion occurs before the closure of the long bones epiphysis), or with a similar pattern to the adult acromegaly (GH hypersecretion after epiphyseal closure). Visual loss, headaches, weight gain and delayed puberty may also occur [55]. Laboratory diagnosis is based on a specific endocrine workup including Insuline-like Growth Factor (IGF)-I and GH levels as well as oral glucose tolerance test and has to be corroborated by a dedicated MRI scan. The mainstay of treatment for GH secreting adenomas is surgical resection whereas in large and invasive tumors surgery can be followed by medical therapy (somatostatin analogues, dopamine agonists or GH receptor antagonists) [62] and/or radiation therapy.

More rare pituitary adenomas include Thyroid-stimulating Hormone (TSH) secreting adenomas, Follicle-stimulating Hormone (FSH)/Luteinizing Hormone (LH) adenomas and non-functioning tumors, the latter accounting for 4–6% of all pediatric adenomas and sometimes associated with hormones deficiency. Non-functioning pituitary adenomas may be observed with regular dedicated MRI when not growing or when discovered as incidental findings. As shown by different cost-effectiveness studies, optimal strategy for pituitary incidental adenomas involves radio-clinical observation with complete endocrine panel and cerebral MRI [63,64]. When surgical treatment is undertaken, endocrine function should be strictly monitored in follow-up: hypopituitarism remains the main long-term postoperative problem, concerning as far as 67% of cases. As for craniopharyngiomas, disease control is problematic: while pediatric patients respond well to surgical management, recurrent tumors are likely to affect negatively the long-term quality of life [65].

### 3.2. Optic Pathway Glioma

Optic Pathways Gliomas (OPG) account for 3–7% of all pediatric gliomas, with a higher incidence in patients affected by Neurofibromatosis type I (NF-1) [6]. These tumors are usually diagnosed in the first decade of life, with a similar distribution between males and females. Histologically they are typically low grade gliomas. Following the 2021 CNS WHO tumor classification, they can be divided into diffuse astrocytoma MTB- or MYBL1-altered, angiocentric gliomas, polymorphous low-grade neuroepithelial tumor of the young or diffuse low-grade glioma MAPK pathway-altered [41]. High-grade gliomas can be present but are less common. They can arise from one optic nerve (25%), involving in 40–75% of cases the optic chiasm. In 33–60% of chiasmatic lesions, a hypothalamic involvement can be found [66]. They can be asymptomatic in 50% of cases. When symptomatic, they present with visual deficits, headaches, endocrine deficit or diencephalic syndrome with behavioural changes and/or HCP. In case of optic nerve compression (most common manifestation) the patient can suffer from proptosis, visual loss or strabismus [67], sign of an early optic nerve involvement. Endocrinologic aberrations usually include GH (40.3%), gonadotropin (20.4%), TSH (13.3%) and ACTH deficiencies (13.3%) [68]. Precocious puberty can be also associated. Imaging workup include both CT and MRI to properly assess bone invasion and optic pathway involvement. In T1 sequences the lesion is iso/hypointense whilst on T2 and on injected sequences it is usually hyperintense. Normally OPG appear solid even though cystic components can be found [69]. Natural history of OPG can range from progression to regression [70]. As repeatedly assessed by the NIH NF-1 Optic Gliomas Task Force, in case of NF-1 and indolent lesions, tumor size follow-up and visual function follow-up is mandatory and if any symptoms or tumor progression are demonstrated, a tailored-made intervention should be considered [71,72]. It should be stressed that OPG are typically slow-growing tumors with slow-declining visual symptoms that often stabilize, allowing for radio-clinical follow-up [67,73].

When symptomatic, management varies greatly and depends mostly on the presence of obstructive HCP and/or visual loss and spans from repeat imaging and visual follow-up to chemotherapy, radiotherapy and surgery. Resective surgery should be offered only for confined optic nerve tumors, in order to avoid contralateral subsequent visual loss. Partial debulking can be considered for mass effect or for concomitant HCP [67], and for extremely rare cases of tumor hemorrhage [74]. Chemotherapy is becoming the main treatment option, considering its low impact on visual function compared to surgery and allowing normal neuropsychological development and neuro-endocrine function [73]. Several regimens can be proposed, with UCSF regimen one of the most used (thioguanine, procarbazine, lomustine and vincristine). New molecular findings such as BRAF, MEK and mTOR inhibitors are being considered as supplementary treatment [16]. For OPG cases in NF new drugs are being tested, such as ERK/MAPK inhibitors [75,76]. Radiotherapy stands usually for tumors refractory to surgery and chemotherapy even though in tertiary referral centers stereotactic radiotherapy, proton beam radiotherapy of radiosurgery are being investigated [66]. As shown by Varan et al. OS and progressive free survival are respectively 83.4% and 54.2% at 10 years, even though surgical and radiotherapy can exacerbate long-term endocrinologic problems [77].

### 3.3. Thalamic Gliomas

Thalamic gliomas account for 1–5% of pediatric intracranial tumors, with a peak of incidence between 8 and 10 years of age and with a similar distribution between males and females [78,79]. Duration of symptoms is directly related to a better prognosis [66]. Anatomically they can be divided into focal, bilateral or diffuse [80]. Bilateral type have a shorter duration of symptoms and carry the worst prognosis. Following 2021 WHO CNS tumor classification if they are high grade they can be associated to H3 K27 alteration, H3 G34 mutation or H3 and IDH-wildtype [41]. Most common symptoms can be related to raised intracranial pressure from obstructive HCP and fluctuation of the level of consciousness (leading potentially to coma), motor/sensory deficits, visual symptoms, endocrine deficits, memory/behavioural problems. On imaging they are formed by solid and/or cystic parts with calcifications. Additional imaging workup include MRI sequences focusing on cell metabolism and perfusion, as well as nuclear medicine exams [81]. Consensus treatment for thalamic lesions is still under debate. In selected cases where a total or subtotal resection is feasible sparing the internal capsule, patients show a better OS [82,83].

A combination of chemotherapy and radiotherapy can be offered in case of high grade variant, recurrent or residual lesion and once a histological diagnosis is made. Chemotherapy is preferred considering its low risk of adverse effect compared to radiotherapy [66] OS varies greatly from low-grade tumors, where the extent of resection become an independent prognostic factor, to high-grade and bilateral thalamic tumors, where OS is as short as 19.5 months [84,85].

In case of rapid radiological change in morphology and appearance, biopsy can be offered in order to maximize adjuvant treatment. The same management can be offered to bithalamic gliomas [78]. Lesions that remain stable and asymptomatic should be followed up with regular clinical assessment and MRI. At the moment there is no consensus and new studies are exploring in detail a more standardized management also based on molecular characteristics [8,79]. 

### 3.4. Pineal Region Tumors

#### 3.4.1. Pineal Parenchymal Tumors

These tumors account for 15–30% of all pineal lesions, they are more frequent in children less than 2 years old, without sex preference and they can be divided into 5 different entities considering their aggressiveness, following the WHO 2021 CNS classification: Pineocytoma, Pineal parenchymal tumor of intermediate differentiation (PPTID), pineoblastoma, papillary tumor of the pineal region (PTPR) and desmoplastic myxoid tumor of the pineal region, SMARCB1-mutant [3,41]. Pineal parenchymal tumors do not present blood markers such as alpha-fetoprotein (α-FP), beta human chorionic gonadotropin (β-hCG) and placental alkaline phosphatase (PLAP) except for synaptophysin in PPTID.

On MRI, parenchymal tumors appear to have heterogenous enhancement on contrast imaging and are restricted in Diffusion Weighted Imaging sequences [12,86]. Cystic components can be associated more frequently with higher grades, but normally are non-evolutive in size [38,87]. Calcification can be found but they are not related to histological type or aggressiveness [88]. Disseminating lesions can occur in pineal or thalamic region, whilst 10–57% of cases show spinal dissemination at the time of the diagnosis: for this reason additional imaging of the spine, endocrine and visual function work-up are recommended [89].

Pineocytomas present well-differentiated cells non-distinguishable from the pineal gland, they are round, usually with spherical-lobular shape, and well separated from adjacent parenchyma. Scarce data are available about this pathology. OS rate at 10-years depends from higher GTR and it is lower for subtotal resection with radiotherapy (84% vs. 17%) [90]. For symptomatic pineocytomas surgery is the first line treatment.

PPTID are an intermediate form of tumors with grade II and/or III characteristics. Histologically they have a positive expression for multiple factors, such as neurofilament, synaptophysin and chromogranin, whose quantity is correlated to their activity and aggressiveness [91]. KBTBD4 gene can be found and new molecular treatments are being studied [92]. On cerebral MRI they tend to have strong contrast enhancement and they have a higher rate of cystic components and calcifications [93]. For PPTID optimal treatment remains still controversial considering the scarcity and rarity of this pathology: A combination of surgery, radiotherapy and chemotherapy seems to be the best first line treatment. Molecular targeted therapy is starting to be considered as a complementary treatment in selected cases [94].

Pinealoblastomas are the most aggressive type of pineal cancer being undifferentiated, harboring the poorest prognosis due to their large dissemination inside and outside CNS [95,96,97]. They account for 40% of pineal parenchymal tumors, they tend to be more common in females and they can be associated with retinoblastomas in trilateral retinoblastoma (concomitant presentation of an intraocular retinoblastoma with a pineoblastoma due to RB1 gene mutation) [98,99]. Markers like chromogranin and synaptophysin can be found in CSF. New molecular classifications are being studied, permitting to find oncogenic drivers and permitting to divide pineoblastomas in 4 different categories: PB-miRNA1 alteration, PB-miRNA2 alteration, PB-MYC amplification/FOXR2 overexpression and PB-RB1 alteration [92]. In MRI T1 and T2 sequences they are heterogeneous, large and poly-lobulated without definite margins. In contrast sequences pinealoblastomas are heterogeneously enhanced. Calcifications, when found, tend to be located peripherally, while cystic components are rare [86]. At 3 years follow-up OS and progression free survival are of 46.7% and 44.4%. A multimodal approach should be proposed by surgical resection and chemotherapy. Radiotherapy can be considered but it is not efficient considering the high risk of metastatic spreading [100].

PTPR are rare tumors classified between WHO grade II and III that arise from subcomissural organ of the posterior commissure and tend to occur in female patients [101]. Morphologically and histologically they are heterogeneous and similar to papillar lesion [9]. On imaging they appear as a large well-circumscribed solid mass (25–40 mm) that has a variable T1 and T2 enhancement and that can have hyperintense T1 signals described as cystic foci [12]. Usually PTPR recurs locally (47% at 6.5 year follow-up) [9] after surgery: radiotherapy and chemotherapy can be offered after resection [102].

Desmoplastic myxoid tumor of the pineal region, SMARCB1-mutant is a new entity proposed in 2021 WHO CNS tumor classification [41].

In general, surgical strategy broadens from stereotactic needle biopsy to resection. In case of benign lesion if the resection is subtotal, radiosurgery can be proposed. In case of resection of an aggressive pineal tumor, adjuvant chemotherapy and radiotherapy are offered to the patient depending on the age of the child [103].

#### 3.4.2. Germ Cell Tumors

Pineal germ cell tumors are predominant in male Asian patients and they are 50% of all germ cell tumors cases [104]. They account for 11.8% of all primary intracranial tumors [104]. Following 2021 WHO CNS classification they are divided into germinomas, choriocarcinomas, teratomas, embryonal carcinomas, yolk sac tumors and mixed germ cell tumors [41]. Oncoproteins should be always searched in serum and CSF: these tumors present elevated values of α-FP, β-hCG and PLAP. Some of them can occur already during fetal development and can be discovered in early life, being non-compatible with it [105].

Germinomas are the most common pineal tumor, capable of spreading all over the CNS. In 8% of cases they are associated to a parallel lesions in suprasellar region, being known as bifocal germinomas [106]. These lesions are normally associated to β-hCG production and a higher level is related to syncytiotrophoblastic cell form and lower OS [98]. On imaging they appear heterogeneous with a solid/cystic aspect and central calcification, being different from pure parenchymal pineal tumors and being iso-hyper intense compared to grey matter [107]. Typically they have a butterfly-shaped form. Germinomas are radiosensitive lesions that effectively respond also to chemotherapy. In case of recurrence, stereotactic radiosurgery may be considered [107,108]. 

Choriocarcinomas are aggressive pineal region tumors, with a low survival rate. They are predominant in pediatric patients with a median OS of 22 months [109]. In the serum and CSF high levels of β-hCG can be found. At imaging they do not present any particularity and it is suggested to complete the work up with spinal imaging. Despite the combination of GTR, chemo- and radiotherapy recurrence rate is high [109].

Teratomas are a common entity in pediatric male patients, with a maximal incidence in new-borns (33% of all intracranial tumors) lowering towards adolescence [110,111]. They are classified as mature, immature or malignant based on the tissue differentiation and they can be totally or partially encapsulated [3]. At imaging they present an important heterogeneity, being polylobate and composed of cystic and calcific regions. In T1 and T2 sequences teratomas have zones of hyperintensity [111]. Surgical resection remains the gold standard and radiotherapy can be considered in malignant forms. α-FP is normally associated to immature forms [111].

Embryonal carcinomas, yolk sac tumors and mixed germ cell tumors present mixed aspect of the tumors mentioned above, their follow up and treatment are tailored to the patient as there are no standardized protocols from large series.

### 3.5. Tectal and Aqueductal Tumors

The terms tectal and aqueductal tumors encompass all the tumors mentioned above and harbour anatomical nuances that have important implications for surgical approaches [87].

Tectal gliomas are a rare pathology that is normally diagnosed incidentally without particular symptoms leading to MRI. A rigorous follow-up is usually offered without the necessity of biopsy [87].

Aqueductal tumors are composed by different types of tumors that manifest mainly with obstructive HCP. In those cases biopsy is often offered when feasible, along with the CSF diversion [87]. At MRI a ballooned aqueduct can be noticed and in 75% of cases tegmental impression is associated [87]. Considering the scarcity of these pathologies the treatment is normally based on personal experience and series. 

## 4. Discussion

Supratentorial midline lesions constitute an heterogenous entity with an extremely different biological and clinical behaviour. From the moment of radiological diagnosis different strategies can be offered to the child and his parents: clinical decisions require a multidisciplinary cooperation between pediatrician, neurologist, neurosurgeon, oncologist, radiotherapist, pediatric neuropsychologist, endocrinologist and ophthalmologist. Compared to the past, parental anxiety has been used as an additional reason to justify treatments. Knowledge of the basic anatomy of the midline structures and their clinical relevance, along with the knowledge of the natural history of such entities, provide a sound guide to all specialists involved in the care of children with supratentorial midline lesions. The ultimate goal is to avoid on one side overzealous futile treatments and parental anxiety, and on the other neglecting clinical risks. In fact, meeting with a specialist neurosurgeon and an appropriate information to parents can change parental anxiety [31].

Despite the variety in their natural history, symptomatic supratentorial midline lesion will usually become clinically manifest with a variable combination of visual, endocrine, behavioural and neuropsychological problems, depending on their location. Such location-specific factors will help guide the therapeutic choice. On the other hand, virtually all of them can induce obstructive HCP regardless of the location in the midline (may it be from obstruction at the foramina of Monro or within the third ventricle or at the aqueduct). HCP in such cases can be life threatening and when present requires CSF diversion, either endoscopic or by shunting [103].

Looking at specific frequent midline lesions:

- Sellar lesions are common in the pediatric population and until visual-endocrine or HCP problems occur imaging follow-up can be continued [63,64].
- For gliomas, some authors suggest an initial close follow up which is subsequently widened (3 months, 6 months and yearly for at least 10-year) paying attention to radiological changes [112]. Malignant transformation is rare and further studies are focusing on evolutive risk factors [8].
- For incidental and asymptomatic colloid cysts follow-up has to be offered with a specialist neurosurgeon and attention should be paid to size changes or new shape irregularities [25,29].
- Pineal lesions harboring abnormal calcification, or large in size can undergo surgery either for biopsy of safe maximal resection: the surgical choice is based on patient’s status, radiologic infiltration of the brainstem, primary dissemination and the presence of focal exophytic components [103]. Surgeon experience and technical knowledge will eventually balance the surgical decision. Exception is made for germinomas where chemotherapy/radiotherapy are still the most efficient treatment [107,113].

Lacking recommendations from an international pediatric consensus, the management on incidental pediatric brain tumors can be influenced by many factors as well as by many specialists involved. In fact lesion-size stability in time remains the main factor leading to a conservative “wait and see” approach [114]. In case of asymptomatic documented growth or parental anxiety the neurosurgeon plays a key role at evaluating risks for malignant transformation, risks for progression to symptoms as well as surgical risks. 

Pediatric neurosurgery is poorly supported by class I evidence despite research in the field: comparative effectiveness research can provide a valid alternative to Randomized Controlled Trials, or can be an alternative research modality in palliative care for seriously ill children [115,116]. The variety of their natural history, the rising prevalence of incidentally found lesions and the lack of data from large series emphasize the need of standardized protocols for managing such tumors and tumor-like lesions.

## 5. Conclusions

Supratentorial midline lesions are rising in prevalence thanks to an easier access to imaging technologies. Their biological and clinical behavior is extremely heterogeneous, ranging from totally indolent and asymptomatic lesions over a life span to life-threatening tumors. Depending on the exact location over the midline, specific clinical and radiological follow-up is suggested for most asymptomatic lesions in the first years and it should involve dedicated specialists. Given the neural structures located in the midline, the clinical assessment is focused mostly on endocrine, visual and neuropsychological function. When considering treatment options versus conservative approach, open discussion about the natural history and parental and children involvement are essential to build a trustworthy relationship over time. Symptomatic lesions are treated according to their specific histological and molecular diagnosis as well as to the extent of visual, endocrine and neuropsychological deficits at individual level. 

## Data Availability

All data are available in the text.

## Abbreviations

CCNST	Childhood Central Nervous System Tumors
OS	Overall Survival
MRI	Magnetic Resonance Imaging
PC	Pineal Cysts
HCP	Hydrocephalus
CT	Computed Tomography
CSF	Cerebrospinal Fluid
WHO	World Health Organization
CNS	Central Nervous System
GTR	Gross Tumor Resection
ACTH	Adrenocorticotropic Hormone
PRL	Prolactine
GH	Growth Hormone
MEN	Multiple Endocrine Neoplasia
CRH	Corticotropin-releasing Hormone
IGF	Insuline-like Growth Factor
TSH	Thyroid-stimulating Hormone
FSH	Follicle-stimulating Hormone
LH	Luteinizing Hormone
OPG	Optic Pathway Gliomas
NF-1	Neurofibromatosis type 1
PPTID	Pineal parenchymal tumor of intermediate differentiation
PTPR	Papillary tumors of the pineal region
α-FP	Alpha-Fetoprotein
β-hCG	Beta human chorionic gonadotropin
PLAP	Placental Alkaline Phosphatase
